# Epigenetic regulation of the human *GDAP1* gene

**DOI:** 10.1016/j.bbrep.2024.101827

**Published:** 2024-09-19

**Authors:** Kaja Karaś, Joanna Pastwińska, Anna Sałkowska, Iwona Karwaciak, Marcin Ratajewski

**Affiliations:** Laboratory of Epigenetics, Institute of Medical Biology, Polish Academy of Sciences, Lodowa 106, 93-232, Lodz, Poland

**Keywords:** GDAP1, CpG island, DNA methylation, Histone acetylation, Epigenetics

## Abstract

Mutations in the ganglioside-induced differentiation-associated protein 1 (*GDAP1*) gene are linked to Charcot–Marie–Tooth (CMT) disease, a hereditary neurodegenerative condition. The protein encoded by this gene is involved in mitochondrial fission and calcium homeostasis. Recently, GDAP1 has also been implicated in the survival of patients with certain cancers. Despite its significant role in specific cellular processes and associated diseases, the mechanisms regulating *GDAP1* expression are largely unknown. Here, we show for the first time that methylation of the CpG island in the proximal promoter of the *GDAP1* gene inhibits its activity. Treating cells with low *GDAP1* expression using methyltransferase and HDAC inhibitors induced the expression of this gene and its encoded protein. This induction was associated with promoter demethylation and increased association of acetylated histones with the *GDAP1* promoter. Thus, we identified a mechanism that could be used to manipulate *GDAP1* expression.

## Highlights

• A CpG island was identified in the proximal *GDAP1* promoter.
• *In vitro* methylation inhibits *GDAP1* promoter activity.
• A DNA methyltransferase inhibitor induces GDAP1 expression and decreases methylation of the identified CpG island.
• An HDAC inhibitor increases GDAP1 expression and enhances the association of acetylated histones with the gene promoter.
• Human *GDAP1* is epigenetically regulated through sequences located in the basal promoter.

## 1 Introduction

Charcot–Marie–Tooth (CMT) disease is a diverse group of heritable genetic disorders characterized by motor and sensory neuropathy in the peripheral nervous system (PNS). The various clinical presentations of CMT are distinguished by the mode of inheritance, age of onset, severity, and form of defects (demyelinating, axonal, and intermediate). Symptoms include loss of sensation, weakness and atrophy of muscles, contractures, and, in severe cases, respiratory impairment [1]. Numerous multigenic mutations have been linked to CMT development, and one of the associated genes is the ganglioside-induced differentiation-associated protein 1 (*GDAP1*) gene, which encodes an integral protein of the outer mitochondrial membrane [2]. Point mutations and truncations in *GDAP1* are responsible for various forms of CMT: CMT4A1 (demyelinating subtype recessively inherited), (AR)-CMT22 (axonal-recessive form), CMTRIA3 (recessive subtype), and CMT2K4 (dominant subtype) [1,3]. The GDAP1 protein is composed of two typical glutathione-S-transferase (GST) domains at the N- and C-termini that are characteristic of proteins involved in the biosynthesis of gangliosides, sialic acid-containing glycosphingolipids, two alpha loops, a transmembrane domain, and a hydrophobic domain [4]. GDAP1 is considered a key regulator of mitochondrial dynamics [2] and is involved in maintaining calcium homeostasis [5] and the cellular redox potential [6]. Recently, an elegant study by Wolf et al. [7] revealed that GDAP1, by interacting with the actin-depolymerizing protein Cofilin-1 and beta-tubulin, affects actin signaling pathways; e.g., when GDAP1 is absent, there is a reduction in F-actin near mitochondria, which hinders the mitochondrial localization of the fission factor dynamin-related protein 1, leading to increased mitochondrial tubularity. Additionally, the loss of GDAP1 disrupts contact sites between mitochondria and the endoplasmic reticulum. These alterations result in decreased mitochondrial calcium levels and shift metabolism toward glutaminolysis. These effects collectively account for the metabolic alterations observed when GDAP1 function is lost [7]. Binieda et al. reported that some mutations in GDAP1 affect its expression and the trans-Golgi network [8]. Moreover, accumulating evidence indicates that GDAP1 expression may be associated with the development of certain cancers and could be a factor influencing patient outcomes [[9], [10], [11], [12]].

Despite the important role of GDAP1 in cellular physiology, little is known about how the gene is regulated at the transcriptional and epigenetic levels, though previous studies have suggested that methylation of the *GDAP1* 5′-flanking region could be a prognostic marker for alcohol dependency [13,14]. Therefore, we aimed to investigate the epigenetic regulation of *GDAP1*. Using in silico approaches, we identified a CpG island located in the proximal promoter region close to the transcriptional start site cluster. This island appeared to be functional, as demonstrated by an *in vitro* methylation assay. Furthermore, we found that in cells with low *GDAP1* expression, the use of methyltransferases and histone deacetylase inhibitors induced *GDAP1* expression. This induction was associated with decreased methylation and increased association of the acetylated forms of histones H3 and H4. Our results show that *GDAP1* is epigenetically regulated, and that its proximal promoter might play an important role in this regulation. These findings might be important for future clinical applications targeting the *GDAP1* gene.

## 2 Materials and methods

### 2.1 Cell lines

The human neuroblastoma SH-SY5Y cell line (with high GDAP1 expression) and the human embryonic kidney HEK293 cell line (with low GDAP1 expression) were obtained from ATCC (Manassas, VA, USA) and cultured under standard conditions at 37 °C in a 5 % CO_2_ atmosphere. The cells were grown in DMEM (PAN Biotech GmbH, Aidenbach, Germany) supplemented with 10 % fetal bovine serum (PAN Biotech GmbH).

### 2.2 Reagents

Trichostatin A (T8552) and 5-aza-2′-deoxycytidine (A3656) were purchased from Merck (Darmstadt, Germany).

### 2.3 In silico analysis of human GDAP1 gene

The CpGplot tool in the European Molecular Biology Open Software Suite (EMBOSS) [15] was used to predict CpG islands in the *GDAP1* gene. Transcriptional start sites (TSSs) were extracted from the DataBase of Transcriptional Start Sites (DBTSS) [16]. Data deposited in the Human Protein Atlas [17,18] were used to analyze the expression of *GDAP1* in various tissues, and methylation data were extracted from the iMethyl database [19].

### 2.4 GDAP1 promoter constructs, transient transfection and reporter assays

All human *GDAP1* promoter constructs were described in our previous study [20]. The reporter constructs were transfected into SH-SY5Y and HEK293 cells using the Fugene HD reagent (Promega, Fitchburg, WI, USA). Twenty-four hours after transfection or after the indicated treatment, luciferase activity in the cell lysates was measured using an Infinite® 200 PRO system (Tecan, Männedorf, Switzerland), with D-luciferin as the substrate (Cayman Chemical, Ann Arbor, MI, USA). Alkaline phosphatase control activity, which was used to assess transfection efficiency, was measured spectrophotometrically at 405 nm.

### 2.5 In vitro methylation of the GDAP1 promoter

To methylate promoter sequences *in vitro*, we extracted the pertinent regions (−9 to +236 and −399 to +236) from phGDAP1(–9/+236)Luc and phGDAP1(–399/+236)Luc plasmids using BglII and *Hin*dIII restriction enzymes (Fermentas/Thermo Fisher Scientific, Waltham, MA, USA). After gel purification, these DNA fragments underwent methylation using SssI DNA methyltransferase (New England Biolabs, Beverly, MA) according to the manufacturer's instructions. Unmethylated and methylated promoter fragments were gel purified and subsequently reintegrated into the pGL3-Basic vector using T4 DNA ligase (Fermentas/Thermo Fisher Scientific), followed by transfection into SH-SY5Y cells. Luciferase activity was then quantified as previously outlined.

### 2.6 Chromatin immunoprecipitation (ChIP)

Chromatin immunoprecipitation (ChIP) was conducted using the EZ-Magna ChIP A/G Kit from EMD Millipore (Billerica, MA, USA) following the manufacturer's protocol. The resulting chromatin was sheared with a VCX-130 sonicator from Sonics & Materials, Inc. (Newtown, CT, USA). The antibodies used were as follows: normal mouse IgG (EMD Millipore) as a control, histone H3ac (pan-acetyl) (pAb) (Active Motif, Carlsbad, CA, USA), and pan-H4Kac (EMD Millipore). To analyze the levels of methylation *in vivo* in HEK293 cells cultured under control conditions or in the presence of 5-aza-2′-deoxycytidine for 5 days, 5 μg of genomic DNA isolated from these cells was subjected to a ChIP assay, which was performed with IgG (EMD Millipore) and 5-methylcytosine (5-mC) (D3S2Z) (Cell Signaling, Danvers, MA, USA). The relative enrichment of the *GDAP1* promoter in the analyzed samples was assessed through real-time PCR with primer pairs specific for the proximal fragment of the *GDAP1* promoter (5′-GCTTTCCAGTCGCAGACC-3′ and 5′-GCCTCTCAGCCATCTTGG-3′), as previously described [20].

### 2.7 RT‒PCR

To assess mRNA abundance, total RNA was extracted from cells using TRI Reagent® (Molecular Research Center, Cincinnati, OH, USA). Five micrograms of RNA was then reverse transcribed using a Maxima First Strand cDNA Synthesis Kit for RT‒qPCR (Thermo Fisher Scientific, Waltham, MA, USA). The resulting cDNA levels were quantified via real-time PCR on a LightCycler 480 (Roche, Basel, Switzerland) using SYBR Green I master mix (Roche) for product detection. The intron-spanning primers used for cDNA detection were described previously: *GDAP1* 5′-GCCTGTCCTTATCCACGG-3′ and 5′-GGCAAGGAGTCAAGCAGC-3′ [20]. The levels of the cognate gene were normalized to the levels of three housekeeping genes: *HPRT1* (5′-TGACACTGGCAAAACAATGCA-3′ and 5′-GGTCCTTTTCACCAGCAAGCT-3′), *HMBS* (5′-TCTCTTATCCCTCACCCTGC-3′ and 5′-ATCACCGCTCTCTGATTTCC-3′), and *RPL13A* (5′-CCTGGAGGAGAAGAGGAAAGAGA-3′ and 5′-TTGAGGACCTCTGTGTATTTGTCAA-3′), as described by Vandesompele et al. [21].

### 2.8 Western blotting

SH-SY5Y or HEK293 cells were lysed using ice-cold RIPA buffer (composed of 50 mM Tris-HCl (pH 8.0), 150 mM NaCl, 0.1 % Triton X-100, 0.1 % SDS, and 0.5 % sodium deoxycholate) supplemented with Halt Protease Inhibitor Cocktail (Thermo Fisher Scientific, Waltham, MA, USA). Western blotting was performed as previously described [22]. The following antibodies were used: anti-GDAP1 (ab194493) (Abcam, Cambridge, UK) and anti-β-actin (#4970) (Cell Signaling Technology, Danvers, MA, USA). Distinct bands were detected using the SuperSignal West Pico Chemiluminescent Substrate (Thermo Fisher Scientific) and captured with a G-Box chemiluminescence imaging system (Syngene, Cambridge, UK).

### 2.9 Statistics

Statistical significance was assessed using analysis of variance (ANOVA) followed by the Student–Newman–Keuls post hoc test with SigmaStat version 4.0 (Systat Software, Inc., San Jose, CA, USA). A p value of less than 0.05 was considered statistically significant.

## 3 Results and discussion

**Fig. 1:**
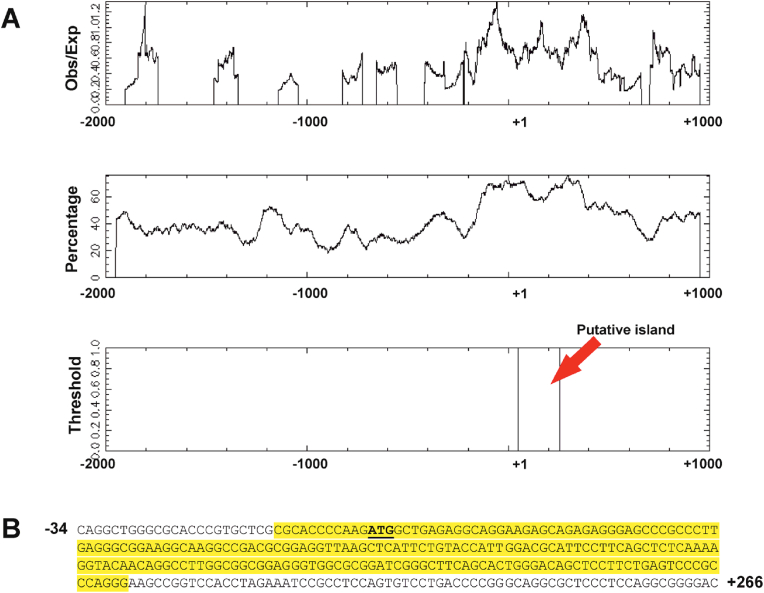
CpG island in the proximal fragment of the human *GDAP1* gene. (A) Results from in silico analysis of the 5′-flanking region of the *GDAP1* gene using CpGplot software. The upper panel shows the ratio of observed vs. expected CpGs, the middle panel shows the percentage of CpG nucleotides across the analyzed region, and the lower panel shows the location of the CpG island. (B) The sequence of the identified CpG island relative to ATG.

**Fig. 2:**
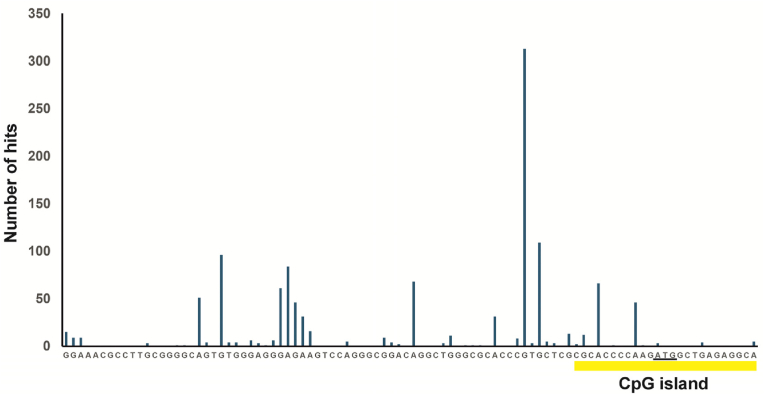
Transcriptional start site cluster located near the identified CpG island in the 5′-flanking region of the *GDAP1* gene. The number of hits starting at each base is shown, with the Y-axis representing the number of transcripts beginning at each base. Data were extracted from the DataBase of Transcriptional Start Sites (DBTSS; https://dbtss.hgc.jp/). The beginning of the CpG island is marked in yellow. The ATG codon is underlined. (For interpretation of the references to colour in this figure legend, the reader is referred to the Web version of this article.)

**Fig. 3:**
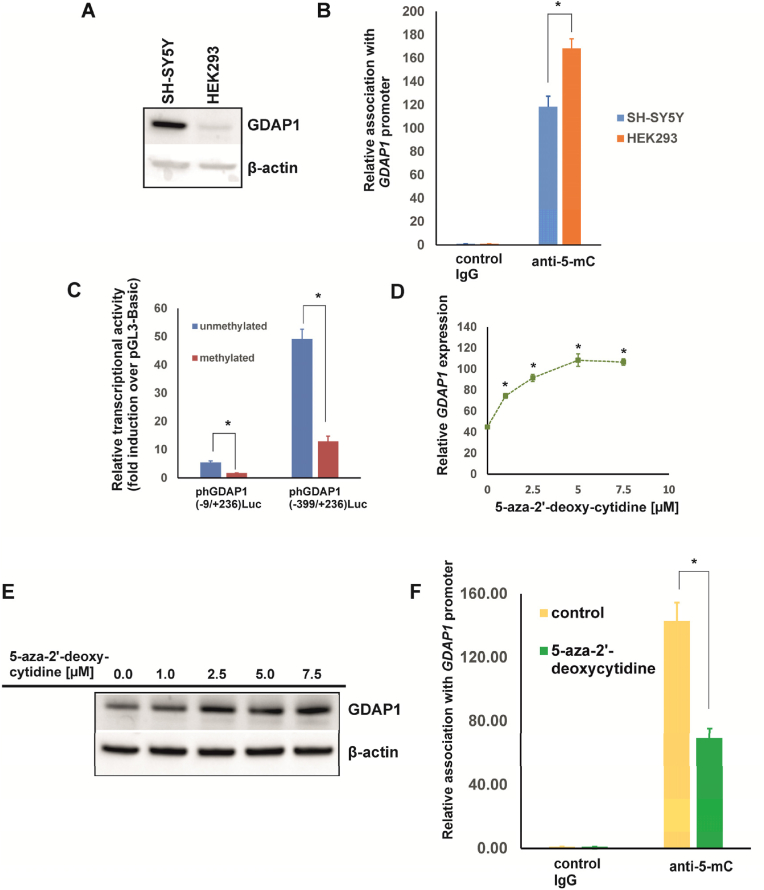
DNA methylation regulates the expression of the human *GDAP1* gene. (A) Expression of GDAP1 at the protein level in the SH-SY5Y and HEK293 cell lines, as determined by western blotting. β-actin was used as a loading control. (B) Comparison of methylation levels of the *GDAP1* promoter in HEK293 and SH-SY5Y cells, as evaluated by chromatin immunoprecipitation performed using control IgG and an anti-5-methylcytosine antibody. The abundance of the *GDAP1* promoter sequence was quantified using quantitative PCR. The results are shown as the means ± SDs; n = 4. ∗Statistically significant difference at p < 0.05. (C) *In vitro* methylation impairs human *GDAP1* promoter activity. SH-SY5Y cells were transiently transfected with luciferase reporter gene constructs that included either methylated or unmethylated *GDAP1* promoter sequences. Luciferase activity was measured and normalized to the activity of a cotransfected SEAP reporter (mean ± SD; n = 4). A statistically significant difference was observed at p < 0.05. (D) Effect of 5-aza-2′-deoxycytidine on the *GDAP1* mRNA level in HEK293 cells. HEK293 cells were cultured in increasing concentrations of 5-aza-2′-deoxycytidine for 5 days, and the expression of *GDAP1* was measured via RT‒PCR (mean ± SD; n = 3). ∗Statistically significant difference at p < 0.05. (E) Effect of 5-aza-2′-deoxycytidine on the GDAP1 protein level in HEK293 cells. HEK293 cells were cultured in increasing concentrations of 5-aza-2′-deoxycytidine for 5 days, and the expression of GDAP1 was determined using western blotting. β-actin was used as a loading control. (F) 5-aza-2′-deoxycytidine leads to demethylation of the *GDAP1* promoter in HEK293 cells, as evidenced by chromatin immunoprecipitation. HEK293 cells were cultured in the presence of 7.5 μM 5-aza-2′-deoxycytidine for 5 days, and chromatin immunoprecipitation was performed using control IgG and anti-5-methylcytosine antibodies. The abundance of the *GDAP1* promoter sequence was analyzed using quantitative PCR. The results are shown as the means ± SDs; n = 4. ∗Statistically significant difference at p < 0.05.

**Fig. 4:**
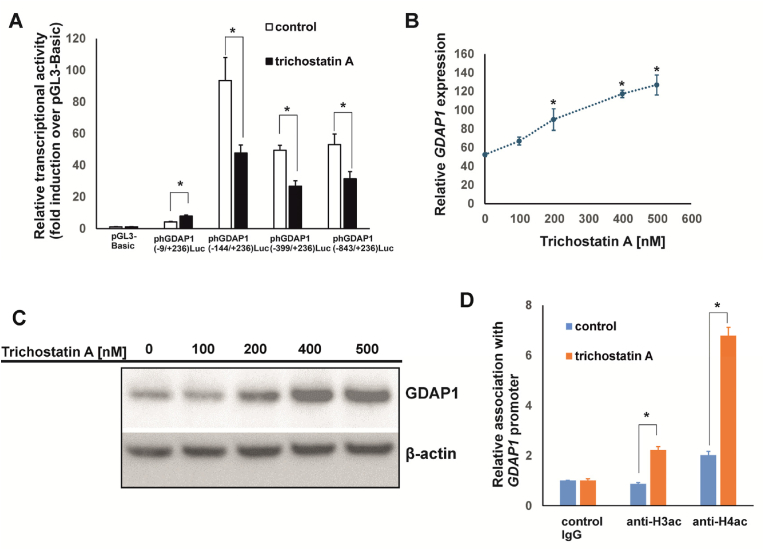
The histone deacetylase inhibitor trichostatin A influences the expression of the human *GDAP1* gene. (A) Identification of the proximal promoter of the *GDAP1* gene as a response element to trichostatin A treatment. HEK293 cells were transfected with various *GDAP1* reporter constructs, and 24 h later, the cells were treated with 400 nM trichostatin A for 48 h. Afterward, luciferase activity was measured and normalized to the activity of a cotransfected SEAP reporter. The results are shown as the means ± SDs; n = 5. A statistically significant difference was observed at p < 0.05. (B) Effect of trichostatin A on the *GDAP1* mRNA level in HEK293 cells. HEK293 cells were cultured in increasing concentrations of trichostatin A for 48 h, and the expression of *GDAP1* was measured by RT‒PCR (mean ± SD; n = 4). ∗Statistically significant difference at p < 0.05. (C) Effects of trichostatin A on the GDAP1 protein level in HEK293 cells. HEK293 cells were cultured in increasing concentrations of trichostatin A for 48 h, and the expression of GDAP1 was determined using western blotting. β-actin was used as a loading control. (D) Trichostatin A led to increased association of acetylated histones H3 and H4 with the *GDAP1* promoter in HEK293 cells, as evidenced using chromatin immunoprecipitation. HEK293 cells were cultured in the presence of 400 nM trichostatin A for 48 h, and chromatin immunoprecipitation was performed using control IgG and anti-H3ac and anti-H4ac antibodies. The abundance of the *GDAP1* promoter sequence was analyzed using quantitative PCR. The results are shown as the means ± SDs; n = 4. ∗Statistically significant difference at p < 0.05.

Despite numerous studies highlighting the role of *GDAP1* in CMT disease [7,[23], [24], [25]] and increasing evidence of its involvement in certain types of cancer [[9], [10], [11]], the mechanisms regulating the expression of this gene have remained largely unexplored. In our previous work, we identified the promoter sequence of the human *GDAP1* gene and discovered that the YY1 transcription factor regulates its expression [20]. We detected the highest expression of *GDAP1* in SH-SY5Y neuroblastoma cells and the lowest in HEK293 cells [20]. This pattern is consistent with those observed in other studies and publicly available databases, e.g. the Human Protein Atlas [17,18], indicating that *GDAP1* expression is highest in the brain and lower in other tissues [24,26] (Supplementary Fig. 1). Thus, we investigated the epigenetic mechanisms underlying the differential expression of *GDAP1* in cells of different origins. We analyzed the *GDAP1* gene sequence from −2000 to +1000 relative to the ATG start codon to identify potential CpG islands [27]. Using CpGplot software, we discovered a single methylation island within the −11 to +197 region (Fig. 1). This island is situated within a cluster of transcription initiation sites (Fig. 2) and includes a previously identified binding site for the YY1 transcription factor [20]. However, notably, data from iMethyl [19] indicate that a 523-base pair CpG island encompasses a larger proximal promoter region (−175/+348) of the *GDAP1* gene. This region was highly methylated in CD4^+^ T cells, monocytes, and neutrophils (Supplementary Fig. 2), which express low levels of *GDAP1*. These findings suggest that methylation at this region could influence the binding of basal and tissue-specific transcription factors associated with *GDAP1* expression. First, we confirmed the expression of GDAP1 at the protein level in SH-SY5Y and HEK293 cells using western blotting. As shown in Fig. 3A, there was a clear difference in protein expression between these two cell lines. ChIP using an antibody specific for methylated DNA revealed that the methylation level in the promoter of the *GDAP1* gene is approximately 30 % higher in HEK293 cells than in SH-SY5Y cells (Fig. 3B). Next, we performed *in vitro* methylation of two *GDAP1* promoter fragments containing the identified CpG island sequence. In both cases, methylation led to a nearly 70 % reduction in promoter activity in SH-SY5Y cells, supporting the hypothesis that this epigenetic modification may regulate *GDAP1* promoter activity (Fig. 3C). Given that promoter methylation is typically associated with inhibited gene expression, we investigated whether this mechanism regulates *GDAP1* expression in cells with low expression levels. We treated HEK293 cells with increasing concentrations of 5-aza-2′-deoxycytidine, a methyltransferase inhibitor [28], for 5 days. Following treatment, we analyzed GDAP1 expression at both the RNA and protein levels. In both cases, we observed a significant increase in GDAP1 expression, which corresponded with a 50 % decrease in the methylation level of the *GDAP1* promoter (Fig. 3D–F). This finding was confirmed using ChIP with an antibody specific for methylated DNA (Fig. 3F). Next, we investigated whether we could increase GDAP1 expression in HEK293 cells using the histone deacetylase (HDAC) inhibitor trichostatin A [29]. First, we examined the responsiveness of the *GDAP1* promoter to this HDAC inhibitor. Deletion analysis revealed that fragments containing the basal promoter (−9/+236) presented a twofold increase in activity in response to trichostatin A. However, longer promoter fragments presented inhibited activity, suggesting increased binding of repressor(s) following trichostatin A treatment, similar to what is observed in the *SL*C*2A5* gene (Fig. 4A) [30]. Despite this unexpected result, we tested whether trichostatin A affects *GDAP1* mRNA and protein levels in HEK293 cells. The RT‒PCR and western blotting results demonstrated that trichostatin A increased *GDAP1* expression, and the highest induction was observed with a 400 nM HDAC inhibitor (Fig. 4B‒C). This increase was associated with increased binding of acetylated histones H3 and H4 to the proximal promoter region (Fig. 4D), which in turn likely increased the availability of the transcription apparatus to the gene promoter [31]. Interestingly, the association of these histones with the *GDAP1* gene promoter in intact cells was very low, which further suggests that this region is not transcriptionally active in HEK293 cells (Fig. 4D).

Mutations in the *GDAP1* gene lead to CMT disease [7]. To date, no mutations within the promoter region have been identified [32,33]. However, regulatory mutations or environmental factors [34] may affect the *GDAP1* expression level [8], contributing to the clinical variability and penetrance of the disease [35]; our finding could thus have clinical significance for potential increase in GDAP1 expression in some CMT patients. Recent findings have highlighted the importance of understanding *GDAP1* regulation, especially in light of its association with cancer patient survival. In acute myeloid leukemia, high *GDAP1* expression is strongly correlated with poor survival [11]. Downregulation of *GDAP1* using specific short interfering RNAs (siRNAs) combined with cytarabine treatment inhibited proliferation and induced apoptosis in the THP-1 cell line [11]. Conversely, in pancreatic ductal adenocarcinoma, high *GDAP1* expression has been linked to increased patient survival [9]. These contrasting outcomes underscore the complex regulatory roles of GDAP1 across different diseases and emphasize the need for further research into its regulatory and therapeutic implications. Interestingly, previous studies have shown that CpG islands in the *GDAP1* gene, located 200 nucleotides from the TSS, are demethylated (with a difference of only 4.9 % or 1.2 % identified between controls and patients) in alcohol-dependent patients, suggesting that they could serve as biomarkers for alcohol dependency [13,14]. However, these studies did not analyze *GDAP1* expression to determine whether these changes were correlated with actual mRNA or protein levels. While we cannot rule out the possibility that some of the cytosines in this region may be methylated, *in vitro* methylation assays have shown that methylation in the −11 to +197 region is crucial for promoter activity (Fig. 3). Other authors have reported an increase in GDAP1 levels in the mouse hippocampus due to cigarette smoking, suggesting that this effect may be associated with methylation, although they did not provide evidence to support this claim [36].

## 4 Conclusions

In the present study, we identified a CpG island in the proximal promoter region of the *GDAP1* gene and confirmed its importance in regulating GDAP1 mRNA and protein expression. Additionally, by employing methyltransferase and HDAC inhibitors, we demonstrated that the basal promoter plays a critical role in the epigenetic regulation of this gene. These findings suggest that targeting the basal promoter could be a viable strategy for potential therapeutic interventions aimed at modulating GDAP1 expression. However, since our analysis was limited to the regulatory sequences near the ATG start codon, we cannot rule out the possibility that distant regions of the *GDAP1* gene are also involved in its epigenetic regulation. Therefore, it would be intriguing to identify these and other potential regulatory sequences using methods such as DNA hypersensitivity assays, as previously performed for other genes, e.g. *ABCC6* [37].

## CRediT authorship contribution statement

**Kaja Karaś:** Writing – original draft, Validation, Methodology, Investigation, Formal analysis, Data curation. **Joanna Pastwińska:** Validation, Methodology, Investigation, Formal analysis, Data curation. **Anna Sałkowska:** Validation, Methodology, Investigation. **Iwona Karwaciak:** Writing – review & editing, Validation, Methodology, Investigation, Data curation. **Marcin Ratajewski:** Writing – review & editing, Writing – original draft, Visualization, Validation, Supervision, Project administration, Methodology, Investigation, Funding acquisition, Formal analysis, Data curation, Conceptualization.

## Declaration of competing interest

The authors declare that they have no known competing financial interests or personal relationships that could have appeared to influence the work reported in this paper.

## Data Availability

Data will be made available on request.
