# Supplementary material for: Epigenetic regulation of the human GDAP1 gene
Source: Biochem Biophys Rep. 2024 Sep 19;40:101827. doi: 10.1016/j.bbrep.2024.101827 (PMC11426145; doi:10.1016/j.bbrep.2024.101827)
Supplement: Multimedia component 1 [file mmc1.docx]

**SUPPLEMENTARY MATERIALS**


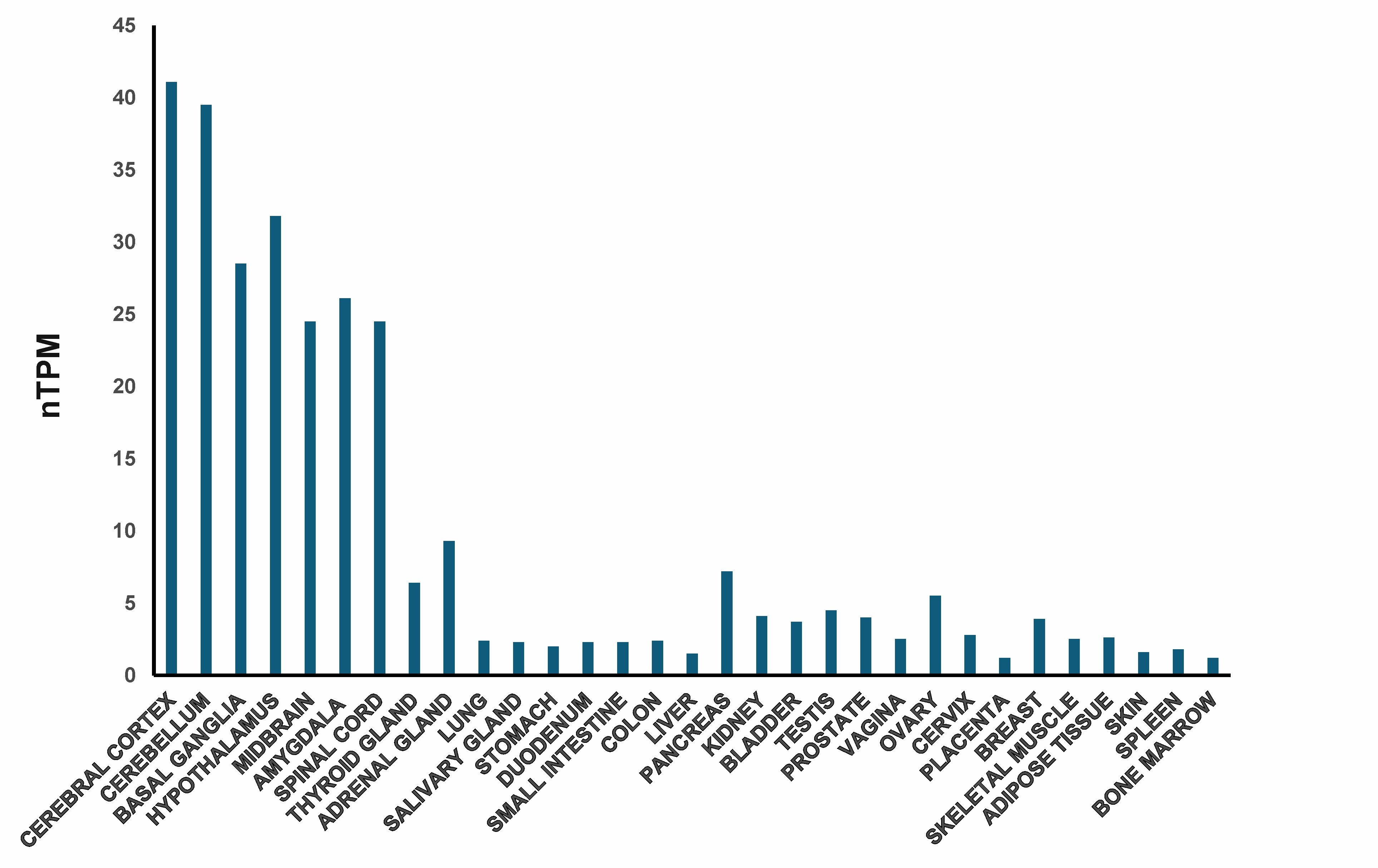


Supplementary Figure 1. Expression level of GDAP1 in cells of different origins. The data are shown as the number of transcripts and were extracted from The Human Protein Atlas (https://www.proteinatlas.org/).


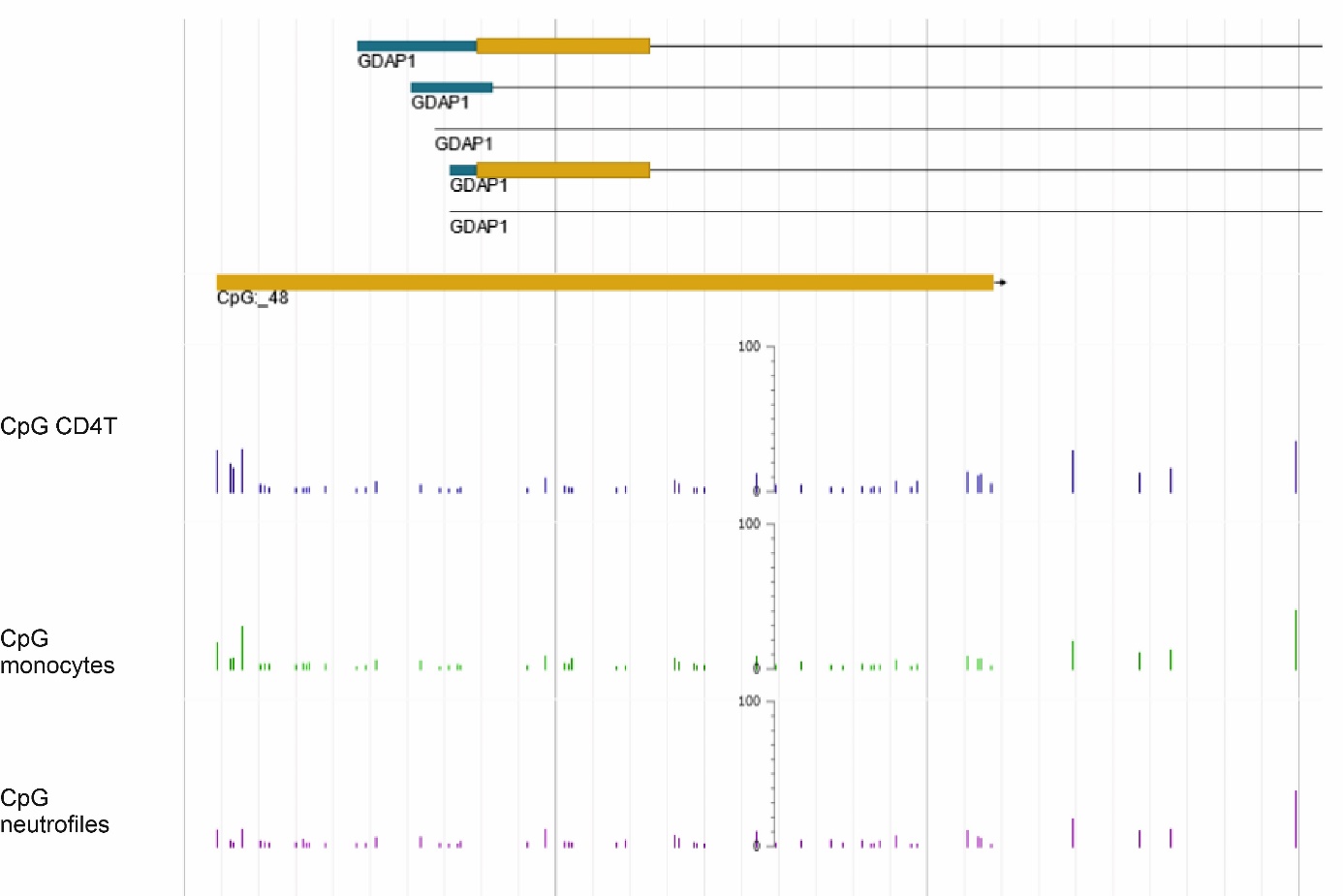


Supplementary Figure 2. Methylation level of the CpG island in the GDAP1 promoter in CD4+ T cells, monocytes and neutrophils. The data were extracted from the iMethyl (http://imethyl.iwate-megabank.org/).

ORIGINAL WESTERN BLOT SCANS

GDAP1


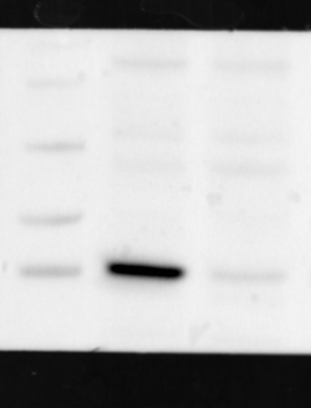


β-actin


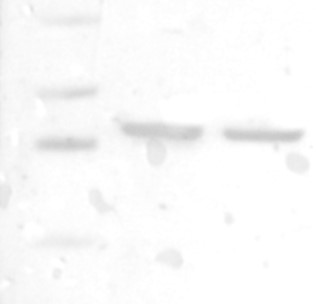


GDAP1


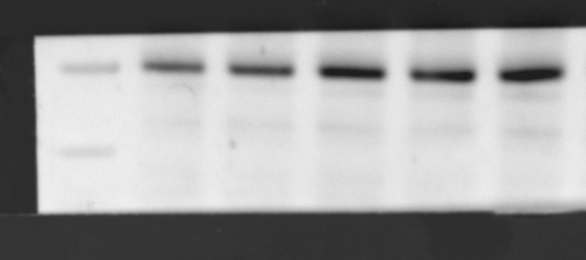


β-actin


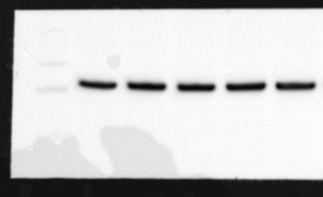


Supplementary Figure 3. Original blot scans for figure 3.

GDAP1


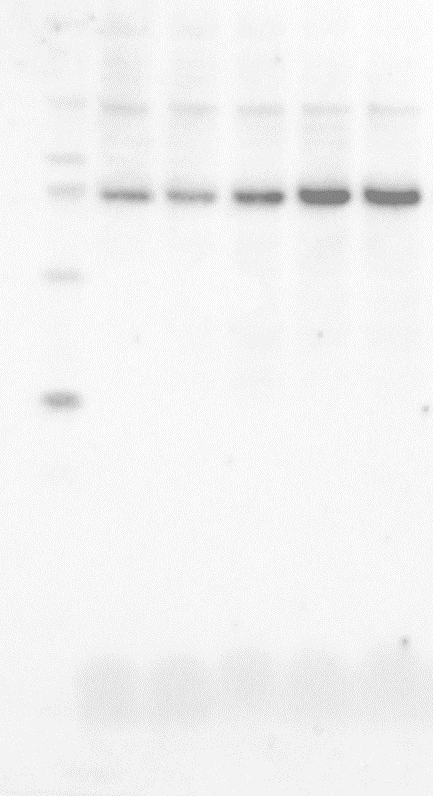


β-actin


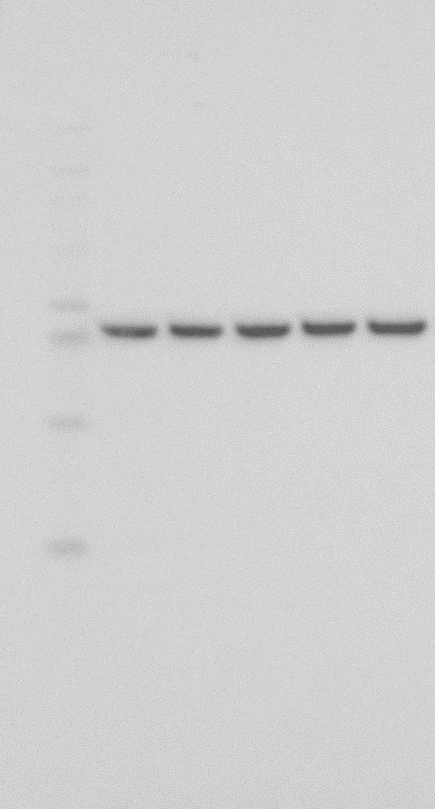


Supplementary Figure 4. Original blot scans for figure 4.
